# Targeting myeloid cells to prevent recurrent stroke in general population: the lesson of hydroxyurea in myeloproliferative neoplasms

**DOI:** 10.1038/s41408-018-0143-y

**Published:** 2018-11-07

**Authors:** Tiziano Barbui, Guido Finazzi, Alessandro M. Vannucchi, Valerio De Stefano

**Affiliations:** 1 FROM Research Foundation, Papa Giovanni XXIII Hospital, Bergamo, Italy; 2 Hematology Unit, Papa Giovanni XXIII Hospital, Bergamo, Italy; 3 CRIMM-Center of Research and Innovation of Myeloproliferative Neoplasms, Azienda Ospedaliera Universitaria Careggi, and Department Experimental and Clinical Medicine, University of Florence, Firenze, Italy; 4 Fondazione Policlinico Universitario A. Gemelli IRCCS, and Istituto di Ematologia, Università Cattolica del Sacro Cuore, Roma, Italy

Recent advances in cell biology have expanded our knowledge on the contribution of myeloid cells, especially leukocytes, to atherosclerosis and arterial thrombosis. Leukocytes, namely monocytes, macrophages, and neutrophils, express and release coagulation and fibrinolytic factors, and interact with the hemostatic system through innate immune functions. As a consequence, inflammatory cell-dependent mechanism and released products are increasingly being considered as potential drug targets for treatment of atherosclerosis, myocardial infarction, and ischemic stroke^1,2^.

The risk of atherosclerosis and thrombosis is increased in clonal hematopoiesis of indeterminate potential (CHIP), a condition defined by the presence of an expanded blood-cell clone harboring somatic acquired genetic variants in persons without other hematologic abnormalities. CHIP strongly associates with increasing age, and this relationship may contribute to increased cardiovascular risk in the elderly. Actually, the presence of CHIP in peripheral-blood cells has been associated with increased frequency of coronary heart disease in humans and with accelerated atherosclerosis in mice^2^.

Chronic Philadelphia-negative myeloproliferative neoplasms (MPN), that include polycythemia vera (PV), essential thrombocythemia (ET), and Myelofibrosis (MF), are neoplastic disorders where clonal hematopoiesis is sustained by three phenotype driver mutations (JAK2V617F, Calreticulin, and MPL) that lead to qualitative and quantitative abnormalities of platelets, leukocytes, and red blood cells; clinically, they are characterized by a marked increase of incident arterial and venous thrombosis, compared to the age-matched general population, that is the major cause of morbidity and mortality^3^. Therefore, MPNs represent a unique clinical model where the anti-thrombotic efficacy of drugs directed against myeloid proliferation might be evaluated. A candidate drug is hydroxyurea (HU), an antimetabolite that prevents DNA synthesis; it was introduced in the therapy of MPNs following the demonstration of its efficacy to reduce the incidence of total thrombosis and in particular of arterial cerebrovascular complications^4,5^. The antithrombotic efficacy of HU is attributed not only to its action in reducing the myeloid proliferation but also to additional mechanisms including qualitative changes in leukocytes, decreased expression of endothelial adhesion molecules, and enhanced nitric oxide generation^3^.

The PRISM Study (Preventing Ischemic Stroke in Myeloproliferative neoplasms) collected retrospective information about 597 patients with MPN of which 270 had presented transient ischemic attacks (TIA) and 327 ischemic stroke (IS); secondary prophylaxis included aspirin, oral anticoagulants, and almost all patients received HU^6^. The baseline blood counts were similar in the two groups, as well as other clinical characteristics apart for arterial hypertension, that was more frequent in IS than TIA, and history of microvascular disturbances that was more common among patients with TIA. Of note, atrial fibrillation was observed in a minority of cases (4% in TIA and 7% in IS) as compared with the general population (11–18 and 20%–30%, respectively)^6^. After one year since the TIA index event, no strokes occurred in the first two years and only 1.24% of cases were recorded after five years; conversely, in patients with a history of IS, the incidence of stroke recurrence was 2.03% and 6.5% after 1 and five years^6^.

We have compared the five years-outcome in the PRISM study with the estimates reported in non-MPN patients with a first TIA^7^ or cryptogenic stroke^8^ (Table 1). Notably, the cumulative incidence of recurrent stroke at five years was much lower in MPN compared to non-MPN population either after TIA and cryptogenic stroke. In the PRISM study, significant predictors of recurrent TIA and IS in multivariate analysis were the same index events (hazard ratio, HR = 2.41 and 4.41, respectively) and the remote history of cerebrovascular TIA episodes (HR = 3.40); furthermore, microvascular disturbances, such as erythromelalgia, scintillating scotoma, pulsatile headache, dizziness, and tinnitus, that are commonly expressed by MPN patients, were independently associated with TIA index events (HR = 2.30). Other prognostic factors of recurrent IS were arterial hypertension (HR = 4.24) and occurrence of IS during the course of MPN (rather than at diagnosis) (HR = 4.27)^6^.

**Table 1:** Comparison of the 5 years-outcomes in different cohorts of patients with cerebrovascular events. Major cardiovascular events are a composite of cardiovascular (CV) death, non-fatal stroke, and non-fatal acute myocardial infarction

Outcomes after 5 years % (95% CI)	Patients			
	TIA Registry	PRISM Study	OXVASC Study	PRISM Study
	Non-MPN patients with TIA or minor stroke	MPN patients with TIA	Non-MPN patients with cryptogenic stroke	MPN patients with ischemic stroke
	*N* *=* *3,847*	*N* *=* *270*	*N* *=* *392*	*N* *=* *327*
Major CV events	12·9 (11·8–14·1)	6·6 (3·1–10·0)	N/R	14·7 (10·1–19·3)
CV death	2·7 (2·2–3·3)	2·1 (0·8–5·0)	10·0 (6·7–13·3)	7·1 (4·5–11·3)
Acute coronary syndrome	1·1 (0·8–1·6)	3·7 (1·8–7·4)	4·2 (2·0–6·4)	1·8 (0·6–4·9)
Ischemic stroke	9·5 (8·5–10·5)	1·2 (0·3–4·9)	23·2 (18·3–28·1)	6·5 (3·9–10·8)
TIA	8·3 (7·4–9·2)	12·0 (8·2–17·2)	N/R	5·8 (3·3–10·1)
Stroke or TIA	16·8 (15·6–18·1)	13·2 (8·5–17·9)	N/R	12·3 (7·8–16·9)

*OXVASC* Oxford Vascular Study^8^*PRISM* Preventing Recurrent Ischemic Stroke in Myeloproliferative Neoplasms^6^, TIA Registry^7^, *N/R* not reported

Notably, after adjustment for sex, age, blood cells values, atrial fibrillation, cardiovascular risk factors, and antithrombotic treatment, cytoreductive therapy with HU emerged as a strong protective factor able to reduce the probability of new IS by 76% (HR = 0.24)^6^. We have speculated that this low rate of recurrences could be attributed to the control of leukocytosis and platelet number by HU and to the improvement of activated MPN clone-derived myeloid blood cells leading to a reduction of accompanying inflammation. It should be emphasized that the myeloid control following therapy with HU has a rapid onset and the blood recovery is relatively rapid when the drug is stopped. HU is the drug of choice for treatment of sickle cell disease, reducing the frequency of painful episodes and the need for erythrocyte transfusions; the long-term efficacy profile is overall favorable without the adverse effects of excessive myelotoxicity, impaired growth and development, altered female fertility, or increased carcinogenicity^9,10^. Concerns regarding the potential leukemogenic effect of HU in MPN patients remained a controversial issue for many years, but recent data have clarified that this potential risk is limited and rarely occurs even after many years of treatment. In a population-based study, the rate of leukemic transformation in a cohort of 11,039 MPN patients diagnosed between 1958 and 2005 was 2.4%, and exposure to HU was not significantly associated with an increased risk of transformation at any cumulative dose level, neither in a crude analysis nor after adjustment for other treatments. Of note, leukemic transformation occurred also in patients never exposed to any cytoreductive agent, and is indeed a risk intrinsic to the MPN disease itself^11^.

In conclusion, from these observations, it would emerge that targeting myeloid cells with HU may reduce the probability of recurrent stroke and one may speculate that this treatment is also indicated in non-MPN general population, particularly in patients who are at high risk for short-term recurrences and in those with high leukocyte counts. This hypothesis deserves to be tested in well-controlled clinical trials.
